# The association of complex liver disorders with HBV genotypes prevalent in Pakistan

**DOI:** 10.1186/1743-422X-4-128

**Published:** 2007-11-27

**Authors:** Saeeda Baig, Anwar Ali Siddiqui, Waqaruddin Ahmed, Huma Qureshi, Ambreen Arif

**Affiliations:** 1 Associate professor, Department of Biochemistry, Ziauddin Medical College, Ziauddin University, Karachi, Pakistan; 2 Associate Dean, Department of Research and Department of Biological and Biomedical Sciences, Aga Khan University, Karachi, Pakistan; 3 Incharge, Pakistan Medical Research Council, Jinnah Postgraduate Medical Center, Karachi, Pakistan; 4 Executive Director Pakistan Medical Research Council, Islamabad, Pakistan; 5 Research Officer, Pakistan Medical Research Council, Jinnah Postgraduate Medical Center, Karachi, Pakistan

## Abstract

**Background:**

Genotyping of HBV is generally used for determining the epidemiological relationship between various virus strains and origin of infection mostly in research studies. The utility of genotyping for clinical applications is only beginning to gain importance. Whether HBV genotyping will constitute part of the clinical evaluation of Hepatitis B patients depends largely on the availability of the relevance of the evidence based information. Since Pakistan has a HBV genotype distribution which has been considered less virulent as investigated by earlier studies from south East Asian countries, a study on correlation between HBV genotypes and risk of progression to further complex hepatic infection was much needed

**Methods:**

A total of 295 patients with HBsAg positive were selected from the Pakistan Medical Research Council's (PMRC) out patient clinics. Two hundred and twenty six (77%) were males, sixty nine (23%) were females (M to F ratio 3.3:1).

**Results:**

Out of 295 patients, 156 (53.2%) had Acute(CAH), 71 (24.2%) were HBV Carriers, 54 (18.4%) had Chronic liver disease (CLD) Hepatitis. 14 (4.7%) were Cirrhosis and HCC patients. Genotype D was the most prevalent genotype in all categories of HBV patients, Acute (108), Chronic (39), and Carrier (53).

Cirrhosis/HCC (7) were HBV/D positive. Genotype A was the second most prevalent with 28 (13%) in acute cases, 12 (22.2%) in chronics, 14 (19.7%) in carriers and 5 (41.7) in Cirrhosis/HCC patients. Mixed genotype (A/D) was found in 20 (12.8%) of Acute patients, 3 (5.6%) of Chronic and 4 (5.6%) of carriers, none in case of severe liver conditions.

**Conclusion:**

Mixed HBV genotypes A, D and A/D combination were present in all categories of patients except that no A/D combination was detected in severe conditions. Genotype D was the dominant genotype. However, genotype A was found to be more strongly associated with severe liver disease. Mixed genotype (A/D) did not significantly appear to influence the clinical outcome.

## Background

HBV is a classical virus that has amazed the researchers and clinicians around the world first with geographic relationship of its genotypes then secondly the association of its different genotypes with a wide spectrum of clinical manifestations. In the recent years, there has been an explosion of knowledge regarding clinical significance of HBV genotypes in terms of clinical outcomes and therapeutic response to antiviral therapy in patients with HBV related severe liver conditions [1]. Approximately 2 billion people in the world are infected by HBV [2], More than 350 million people are chronic carriers of the virus [3] Acute hepatitis of varying severity exists in 95% of children and 2–10 % of adult patients [4]. Overall, less than 1 % of acute infections lead to fulminant hepatitis and death. Approximately 0–10 % of infected adults become chronic carriers of HBV [5,6]. Chronic HBV infection is currently the most common cause of cirrhosis and hepatocellular carcinoma (HCC) in the world. Fifteen to 40% of chronically infected people may develop cirrhosis and HCC, the remaining individuals become asymptomatic carriers. In perinatal transmission, there is a strong chance for the child to become chronically infected compared to the infection acquired during adulthood,(about 10% to 20%) [7,8].

Initially studies on the effect of genotypes on disease progression were reported from South-east Asian countries where HBV is hyperendemic. Since genotypes B and C are most prevalent in this region severity of liver dysfunction was found associated with these genotypes. Patients infected with genotype C were found having higher HBV-DNA levels compared with those infected with genotype B [9-11], A, and D [12], in some studies but not in others [13,14]. Pakistan, according to WHO, falls in the low endemic area of HBV infection with prevalence of 3% infected population. Studies from Pakistan focused more towards the HBV prevalence rate [15,16], epidemiological issues [17], genotyping of most prevalent strain and its genetic variability regarding core region.[18] Since the most prevalent genotype is D [18] coexisting with less prevalent genotype A and A+D less than 20% [19], it was required to investigate the virulence of these genotypes, especially regarding genotype D which has been found as less virulent and genotype of the asymptomatic carriers by earlier studies from south East Asian countries. This research study was conducted to assess the correlation between HBV genotype D, A and A+D and risk of progression to further complex hepatic infections such as chronic, acute, cirrhosis and HCC.

## Results

**Figure 1:**
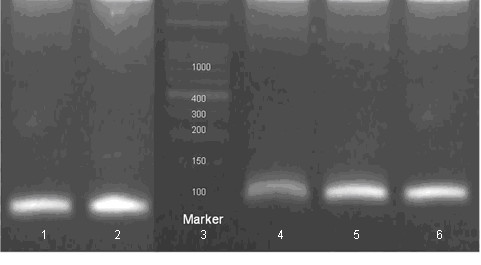
3% agarose gel showing genotype specific bands in patients infected with hepatitis B virus. Lane 1 and 2 show HBV genotype A specific 68 bp band; Lane 3 show 100 bp marker; Lane 4,5 and 6 show HBV genotype D specific 119 bp band.

Two hundred and ninety five HBsAg positive registered patients were selected from the PMRC OPD. Two hundred and twenty six (76.6%) were males, 69 (23.4%) were females (M to F ratio 3.3:1) Table 2. Out of 293, 156 (53%) had Acute (CAH), 71 (24%) were HBV Carriers, 54(18.3%) had Chronic(CLD) Hepatitis, Cirrhosis and HCC patients were 14 (4.7%). Generally, genotype D (Figure 1) was the most prevalent genotype in all categories of HBV patients, Acute (69.2%), Chronic (72.2%), Carrier (74.7%)). Cirrhosis/HCC (62.2%) were HBV/D positive. Genotype A was the second prevalent with 28 (13%) in acute cases, 12 (22.2%) in chronics,14 (19.7%) in carriers and 5 (37.7.8%) in Cirrhosis/HCC patients. Mixed genotype A+D was found in 20 (12.8%) of Acute patients, 3 (5.6%) of Chronic and 4(5.6%) of carriers (Table 3).

**Table 2:** Genotype distribution according to disease and gender

**Diagnosis/Genotype**		**Male n = 226(76.6%)**	**Female n = 69(23.4%)**	**Total (n = 295)**
**Acute (CAH)**		**124**	**32**	**156(52.9%)**
**Genotype**	D	88 (71.0%)	20 (62.5%)	108 (69.2%)
	A	23 (18.5%)	5 (15.6%)	28 (18.0%)
	AD	13 (10.5%)	7 (21.9%)	20 (12.8%)
**Chronic (CLD)**		**43**	**11**	**54(18.3%)**
**Genotype**	D	30 (69.8%)	9 (81.8%)	39 (72.2%)
	A	10 (23.3%)	2 (18.2%)	12 (22.2%)
	AD	3 (6.9%)	-	3 (5.6%)
**Carrier (asymptomatic)**		**48**	**23**	**71(24%)**
**Genotype**	D	38 (79.2%)	15 (65.2%)	53 (74.7%)
	A	6 (12.5%)	8 (34.8%)	14 (19.7%)
	AD	4 (8.3%)	-	4 (5.6%)
**Cirrhosis/HCC**		**11**	**3**	**14(4.7%)**
**Genotype**	D	7 (63.6%)	1(33.3%	8 (57.14%)
	A	4(36.3%)	2 (66.6%)	6 (43.8%)
	AD	-	-	-

**Table 3:** Primer sequences used for HBV genotyping by nested PCR (position, specificity, and polarity). An "M " represents a nucleotide that could be either an A or a C; a "Y" represents a nucleotide that could be either a C or a T. nt, nucleotide.

	Primers	Sequence	Gene/CDS	Product
Step-one PCR	P1b universal, sense)	5'-TCA CCA TAT TCT TGG GAA CAA GA-3'	nt2823-2845,	1065 bp
	S1-2 universal, antisense)	5'-CGA ACC ACT GAA CAA ATG GC-3'	nt685-704,	
Step-two PCR	B2 sense	5'-GGC TCM AGT TCM GGA ACA GT-3'	nt67-86, types A specific, to E	
Mix A	BAIR antisense	5'-CTC GCG GAG ATT GAC GAG ATG T-3'	nt113-134, type A specific,	68 bp
	BBIR antisense	5'-CAG GTT GGT GAG TGA CTG GAG A-3'	nt324-345, type B specific,	122 bp
	BCIR antisense)	5'-GGT CCT AGG AAT CCT GAT GTT G-3'	nt165-186, type C specific,	281 bp

## Discussion

This is the first study from Pakistancomparing the clinical outcome of HBV-related liver disease in patients infected with different HBV genotypes using a PCR based method. Out of 295 HBsAg positive registered patients, 225 (77%) were males, and 69 (23%) were females (M to F ratio approximately 3.3:1) who were suffering from various liver conditions were genotyped (Table 2). Genotype A, D and both A and D (figure 1) were present in all categories of HBV patients but the most prevalent genotype was D in all conditions except cirrhosis in which genotype A was dominant. This pattern of genotype prevalence in Pakistan is in accordance with studies from South East Asia, especially countries sharing borders with Pakistan such as Afghanistan, Iran and India having dominance of genotype D. Chattopadhyay [21] reports, in a similar study from India that Genotypes D and A were present in all CLDB patient categories and genotype D was dominant. Generally, apart from HBV genotype B and C there is lack of information about the clinical course of HBV infected patients with other genotypes.

The clinical significance of different HBV genotypes has become increasingly recognized in patients with acute and chronic infection [22]. Genotyping of Acute patients in this study revealed genotype D(69.2%) as most prevalent genotype, whereas, A and AD were 18.0% and 12.8% respectively. There is paucity of information on the correlation between HBV genotypes and outcome of acute HBV infection. Acute HBV infection in Europe in earlier studies was found to be associated with genotype D [23,24], but recently it has been demonstrated that A is the most prevalent amongst the patients with acute Hepatitis B [25]. Similarly a study from Japan demonstrates HBV genotype A in patients with acute hepatitis B where the most prevalent genotype Band C lead to chronic infection [26]. This leads us to believe that different genotypes may be associated with different rates of progression from acute to chronic HBV infection. If we conclude that these reports are possibly not reflecting the geographical difference but instead are showing the most prevalent genotype as most virulent as in the case of Europe then why is genotype A more common in Acute infection in Japanese patients when it should be B or C.

In Chronic infection levels of viremia are generally low. The strong determinant of chronicity is age at the time of infection. Long-term prognosis is poorer among HBeAg-negative individuals compared to their counterparts who are HBeAg-positive. In this study 18.4% had Chronic (CLD) Hepatitis. Out of 54 chronic patients, 72.2% had genotype D, 22.2% had A and 5.6% had AD. This is in contrast to a study by Mayerat et al. [23] which suggested that the chronic infection by HBV could be more frequently associated with genotype A than genotype D. Whereas, genotype D was more prevalent among patients with resolving acute HBV infection, suggesting that HBV genotype D was associated with a lower rate of chronic HBV infection; however, Erhardt et al.'s [27] study on comparison of HBV genotypes A and D report that the rate of interferon-induced HBeAg seroconversion was lower among patients with genotype D than among those with genotype A (6% vs. 37%), whereas, the influence of these genotypes on acute HBV infection was inconclusive. HBV genotype D has also been described as the genotype of intravenous illicit drug users [28].

During the Carrier state, low HBsAg levels are marked by HBeAg negativity with anti-HBe positivity, low HBV DNA level and repeatedly normal ALT. In this study the highest prevalence of Genotype D was found in HBV asymptomatic carriers (74.7%). Borchani [29] also reports that Genotype D as the most prevalent amongst theie Asymptomatic Carrier patients (67.2%). A study in Italy has found that carriers of HBsAg who are symptom-free and whose liver function tests are normal have an excellent prognosis and the risk of hepatocellular carcinoma was low over the mean follow-up period of about 30 years [30]. The prognosis of the inactive carrier is generally good and well supported by long-term follow-up studies [31-33]. An estimated 20% to 30% of HBsAg carriers may develop reactivation of hepatitis B with elevation of biochemical levels, high serum DNA level with or without sero-reversion to HBeAg. Recurrent episodes of reactivation or sustained reactivation can occur and contribute to progressive liver disease and decompensation especially, in the immunosuppressed individuals. Frequently, HBV reactivation is usually asymptomatic, but it may mimic acute viral hepatitis [34].

Cirrhosis and Hepatocellular Carcinoma (HCC) are two major long-term complications of chronic HBV infection which developed in 14 of our patients (11 males and 3 females). Five had developed cirrhosis and 9 hepatocellular carcinoma. Chronically infected subjects have a 100 times increased risk of hepatocellular carcinoma compared to non-carriers. HBsAg positivity increases risk of developing HCC by 10 folds and HBeAg positivity by 60 folds, whereas, a detectable HBV DNA level yields a 4 fold increased risk of HCC [35]. Regarding the genotype distribution in this study HBV genotype D was most prevalent among the HCC patients and genotype A in cirrhosis patients. Four out of five Cirrhosis patients in this study had HBV genotype A. A similar study from Spain [36] reports genotype A as more virulent because earlier the core antigen seroconversion rates were similar with genotypes A and D, but sustained biochemical and virological remission was not common with patients with genotype A who had HBeAg seroconversion. These patients had also higher rates of HBsAg clearance. These results indicate that HBV genotype A is associated with more marked ALT elevation, a higher rate of HBeAg positivity and presence of liver cirrhosis.[37]. Thus, it would be interesting to speculate that HBV genotype A could indeed be more prone to chronic infection than genotype D [38].

Among the nine patients in this study who developed HCC two were females and seven were males. Age ranged from 35 yrs to 68 yrs. Genotype D being most prevalent was present in 7(77.7%) patients, whereas genotype A in two patients, who developed HCC from chronic infection. Out of 5 with genotype D, five males, age range 50 yrs to 68 yrs, were long time carriers (fifteen years after suffering from HBV infection) and the rest of the two, one male and other female were in the age range 35 yrs and 45 yrs. Thakur et al. [39] also concluded that genotype D may predict the occurrence of hepatocellular carcinoma in young Indian patients. Several studies have indicated that older age (> 45 years) is an important determinant of HCC. Having a first degree relative with HCC, the presence of cirrhosis, and reversion activity, all contribute to HCC development [40,41]. In this study, all patients with HCC were above 50 years of age but among the 5 patients who developed cirrhosis the age ranged from 22 years to 60 years. Kumar et al [37], also reports cirrhosis of liver to be more frequently in patients aged 25 years and above.

Generally, all categories of liver diseases showed male dominance, especially in HCC it is well documented. HCC incidence is three to six times higher in males than in females, suggesting a tumorigenic effect of androgens [42,43]. The 5 patients who developed cirrhosis from chronic condition were one female and 4 males again showing the predominance of male gender. In an experimentally induced carcinomas spontaneous neoplasms occurred at a higher rate in male rats and mice [44]. The studies on estradiol showed not only suppressive effect of estradiol on chemical hepatocarcinogenesis in rats [45] but also its cytoprotective effect against hepatocyte injury [46]. Thus it can be concluded that a better understanding of the biological mechanisms underlying the gender-associated differences observed in chronic HBV infection may provide valuable information on more effective treatment modalities in liver disease in both males and females [47].

Combination of AD exist where ever genotype A and D are prevalent such as India [39], Italy [48] etc. but none of studies report any virulence associated to it. In this study though AD combination was present in 12% of acute patients, 5.6% of chronic patients and 5.6% of carriers, but none of the cirrhosis or HCC patients had combination of AD.

Gerner et al. [49] observed that patients who were infected with HBV genotype A, after treatment with interferon and a relapse, had a switch of the genotype from HBV genotype A to D, whereas, Chen [50] observed that the dominant-genotype C changed to genotype Ba after anti-HBV e antigen (anti-HBe) seroconversion. Originally HBV/Ba (B2) co-existed as a minor population with HBV/C during the early course of acute HBV infection and then emerged and gradually became the dominant genotype. In our patients since the genotype was not checked before they developed severe conditions therefore, it can not be ascertained whether there was a switch of genotype. The mechanism for this recombination and switching remains enigmatic but this could be a possibility that long term sequelae could lead to reorganization of nucleotides. It is, hence, better advised that all HBV infected patients, regardless of status, should get screened after every 6 months with alpha-fetoprotein (AFP) and liver sonogram.

## Conclusion

Genotyping of HBV may remain a research tool unless we prove that it can predict the risk of adverse outcomes (fulminant disease, cirrhosis, HCC) or can influence decision-making in managing these conditions. Studies so far around the world have lead us to believe that different genotypes may be associated with different rates of progression from acute to chronic HBV infection. However, differences in host and environmental factors make it difficult to extrapolate findings from one geographical region to another. Therefore, larger, in-depth longitudinal prospective studies are necessary, in various regions of the world, that could provide more information on the relationship of HBV genotypes to the severity of liver disease and thereby clinical outcome.

## Methods

### Selection of subjects

HBsAg positive 295 registered patients wereselected. irrespective of age and gender, with HBV-related liver disease such as Acute, Chronic, Cirrhosis, HCC, attending the outpatient Department of Pakistan Medical and Research Council(PMRC), Gastroenterology unit JPMC during the years 2006 to 2007, were selected for the study. All patients were HBV positive, registered patients Clinical data were retrieved from medical records, as were laboratory test results including hemogram, liver function tests, coagulation profile, and findings at abdominal ultrasonography, upper gastrointestinal endoscopy and liver biopsy. Liver cirrhosis and HCC were diagnosed either on the basis of histology, or on a combination of radiological, endoscopic and laboratory data. Serum samples were collected from the study patients when they came for the follow-up. A written as well as verbal informed consent was taken from each subject; however, in case of patients who were under the age of 18, parental consent was obtained. Prior to this approval of ZMUH ethical review committee was obtained. The following inclusion and exclusion criteria were applied while selecting patients for this study:

### Inclusion Criteria

All healthy and diseased HbsAg positive individuals (healthy carriers, acute hepatitis, chronic hepatitis).

### Exclusion Criteria

Patients with other hepatic viral markers (A, C, D, E) with HBV were excluded.

### DNA extraction

DNA extraction from serum was done by DNA extraction kit (Promega Minipreps). The HBV genome was amplified by nested PCR using the universal primers (P1 and S1-2) for the outer primers, followed by two different mixtures containing type-specific inner primers as described above.

### PCR Amplification

A modified version of nested PCR developed by Naito et al. [20] was followed. The sequences of PCR primers(supplied by Gene Link USA) used in this study were in the form of pairs step one and step-two designed on the basis of the conserved nature of nucleotide sequences in regions of the pre-S1 through S genes, irrespective of theHBV genotypes (Figure 1). The HBV genome was amplified by sequence specific PCR using the universal primers (P1 and S1-2) for the outer primers, followed by two different mixtures containing type-specific inner primers as described in Table 1.

**Table 1:** Diagnosis according to gender in different categories of patients

Diagnosis	Male (n = 226) **(76.6%)**		Female (n = 69) **(23.4%)**		Total (n = 295)	
	
	Number	Percent	Number	Percent	Number	Percent
Acute (CAH)	124	54.86	32	46.3	156	52.7
Chronic (CLD)	43	19.0	11	16.0	54	18.2
Carrier	48	21.2	23	33.3	71	24
Cirrhosis/HCC	11	4.8	3	4.3	14	4.7

### Primer sequence and detail

#### Step one PCR

The first PCR was carried out in a tube containing 50 μl of a reaction buffer made up of the following components: 50 ng each of the outer primer, a 200 μM concentration of each of the four deoxynucleotides, 1U of Taq DNA polymerase (Perkin-Elmer, Norwalk, Conn.), and 1× PCR buffer containing 1.5 mM MgCl_2_. The extracted DNA was given an initial 10 min incubation at 95°C for a hot start reaction. After 10 min the PCR program was paused to dispense the master-mix in all tubes. The thermocycler (GeneAmp PCR system 2400,9600, and 9700A; Perkin-Elmer) was programmed to first incubate the samples for 10 min at 95°C, followed by 35 cycles consisting of 94°C for min., 94°C for 20s, 55°C for 20s, and 72°C for 1 min. with a final extension of 5 min. at 72°C and 4°C.

#### Step two PCR

Two second-round PCRs were performed for each sample, with the common universal sense primer (B2) and mix A for types A through C and the common universal antisense primer (B2R) and mix B for types D through F.A 2.5 μl aliquot of the first PCR product was added to two tubes containing the second sets of each of the inner primer pairs, each of the deoxynucleotides, AmpliTaq Gold DNA polymerase, and PCR buffer, as in the first reaction. These were amplified for35 cycles with the following parameters: preheating at 94°C for 3 min, 15 cycles of amplification at 94°C for 20s, 58°C for 30s, and 72°C for 40s, and an additional 20 cycles of 94°C for 30s, 60°C for 30s, and 72°C for 45s with an extension of 7 min at 72°C and incubation at 4°C. Genotypes of HBV for each sample were determined by identifying the genotype-specific DNA bands. The two different second-round PCR products from one sample were separately electrophoresed on a 3% agarose gel,(1% agarose plus 2% Nusieve Agarose) stained with ethidium bromide, and evaluated under UV light. The sizes of PCR products were estimated according to the migration pattern of a 50-bp DNA ladder (Pharmacia Biotech, Uppsala, Sweden).

## Competing interests

The author(s) declare that they have no competing interests.

## Authors' contributions

SB, AAS and WA designed the Research project. SB did all the bench work. SB and AAS wrote the manuscript. WA, HQ and AA clinically assessed the patients prior to selection and also helped in the clinical data retrieval from the medical records (all were their registered patients).
